# Landscape configuration affects herbivore–parasitoid communities in oilseed rape

**DOI:** 10.1007/s10340-018-0965-1

**Published:** 2018-02-24

**Authors:** Josef S. Berger, Klaus Birkhofer, Helena I. Hanson, Katarina Hedlund

**Affiliations:** 1 Department of Biology, Lund University, Sölvegatan 37, 223 62 Lund, Sweden; 2 Centre for Environmental and Climate Research, Lund University, Sölvegatan 37, 223 62 Lund, Sweden; 3 Department of Ecology, Brandenburg University of Technology Cottbus-Senftenberg, Konrad-Wachsmann-Allee 6, 03046 Cottbus, Germany

**Keywords:** Landscape configuration, Community composition, Web asymmetry, Herbivore community, Parasitoid community, Oilseed rape

## Abstract

It is crucial to consider the effects of large-scale drivers on species presences and ecological interactions to understand what structures communities. In our study, we investigated how the species composition and the potential interaction networks of herbivore and parasitoid communities in oilseed rape fields are affected by agricultural landscape characteristics. Insect communities of 26 winter oilseed rape fields in southern Sweden were captured in water traps over a continuous time span of 30 ± 2 days. In total, 31% of the variation in the composition of herbivore host communities was explained by a combination of the surrounding oilseed rape area in the study year and the previous year and distance to the nearest forest. The oilseed rape area in the study year and distance to forest also explained 14% of the variation in the composition of parasitoid communities. Distance to the nearest forest together with the area of oilseed rape in the previous year explained 45% of the variation in asymmetry of interaction webs. These results indicate that several measures of landscape configuration are important both for the composition of host and parasitoid communities and also for the structure of interaction networks. Our results support the view that it is an appropriate strategy to cultivate oilseed rape in landscapes that are far away from forests, in order to minimize recolonization by pest species and at the same time to attract parasitoid species from the open landscape.

**Electronic supplementary material:**

The online version of this article (10.1007/s10340-018-0965-1) contains supplementary material, which is available to authorized users.

## Key messages

- Landscape effects on parasitoids of oilseed rape pests have hitherto mainly been studied on a few species.
- We tested the hypotheses that several landscape variables affect the species composition of both parasitoid and host communities and their interactions.
- Distance from the site to forested land, and oilseed rape area within a 1 km radius, was important predictor of variation in community composition of both trophic levels.
- Host–parasitoid interaction networks become more asymmetric with increasing distance from forests due to relatively fewer pest than parasitoid species.
- Consequently, we predict that growing oilseed rape farther away from forests will attract fewer host species and more species of their natural enemies.

## Introduction

Oilseed rape (OSR) is a suitable habitat for many herbivorous insect species of which some cause considerable economic losses, while others are of little or no economic importance (Alford et al. 2003). In Europe, herbivores in OSR are attacked by at least 80 species of parasitoid Hymenoptera, while only 12 of them are considered to be economically important as biological control agents (Ulber et al. 2010). In intensively managed agricultural landscapes, the presence of hosts and their parasitoids may depend on management strategies and landscape characteristics. Previous research in OSR habitats has mostly focused on single or few target species with high economic impact, such as the rape pollen beetle *Brassicogethes aeneus* and its parasitoids (Thies and Tscharntke 2010; Rusch et al. 2011, 2013; Schneider et al. 2015). However, despite the presence of a diverse herbivore (Hiiesaar et al. 2003; Vaitelytė et al. 2013; Metspalu et al. 2014) and parasitoid community (Tarang et al. 2004; Nerad et al. 2011) in oilseed rape, our knowledge about landscape effects on multiple species in this ecosystem is limited to a few studies (Zaller et al. 2008, 2009; Frank et al. 2010). To fully understand processes that structure these invertebrate communities, we need to take into account not only traditional components of biodiversity such as the total number of species, but also species identity and community composition (Symstad et al. 1998; Emery and Gross 2007) and the ecological interactions between these species (Tylianakis et al. 2010; Miranda et al. 2013).

Landscape characteristics influence invertebrate communities in agricultural crops in various ways (Tscharntke et al. 2007). Landscape complexity for example enhances species richness, abundance or fecundity of parasitoids (Bianchi et al. 2006; Rusch et al. 2010). Parasitoids of the rape pollen beetle also have higher parasitism rates in structurally complex as opposed to structurally simple landscapes (Thies and Tscharntke 1999). The area covered with potential host crops affects agricultural pest species and their parasitoids, but it is uncertain whether their abundances are affected positively or negatively (Rusch et al. 2010; Veres et al. 2013). For pollen beetle larvae in OSR crops, some studies reported a negative effect of crop area on parasitization rate by *Tersilochus heterocerus* (Schneider et al. 2015), while other studies report a positive (Zaller et al. 2009) or no effect (Hanson et al. 2015b). Zaller et al. (2008) and Frank et al. (2010) reported that abundances of adult pollen beetles and of stem weevil larvae are negatively correlated with OSR crop area in the surroundings, but positively correlated with woody areas. Herbivore communities in OSR are complex, and it is unlikely that all species respond in the same way to such landscape characteristics. For example, specialist and generalist species may respond differently to landscape characteristics, since specialists that hibernate in the crop fields need to cope with crop rotation: winter OSR is commonly grown in rotation with cereals and sometimes with root crops (winter wheat, spring barley, sugar beet, oats) in Europe (Williams 2010).

Agricultural fields can be seen as potentially hostile environments for the long-term survival of parasitoids due to intensive management (Hanson et al. 2015a) and temporal reallocation of habitats (Thies and Tscharntke 2010). In contrast, non-crop habitats are more constant in time and provide important life-supporting functions to insects (Rusch et al. 2010). Pollen beetles, for example, are known to hibernate in forests (Nilsson 1989), and their abundance is positively related to the proportion of woody areas around focal OSR fields (Zaller et al. 2008) and the landscape configuration of the previous year oilseed rape fields around overwintering sites (Rusch et al. 2012). The ground litter of both coniferous and deciduous forests has also been documented as hibernation site for a number of other herbivores that occur in OSR, for example the cabbage stem weevil *Ceutorhynchus pallidactylus* and several *Phyllotreta* species (Günthart 1949), as well as the tarnished plant bug *Lygus rugulipennis* (Rämert et al. 2005). For the parasitoids in this system, there are far less indications that the presence of woody areas might be of importance, except *Trichomalus perfectus* (parasitoid of *C. obstrictus*) and some other pteromalids that are assumed to hibernate on coniferous trees (von Rosén 1964). Several parasitoid species that are specialized on OSR pests (e.g. *Phradis interstitialis*, *P. morionellus*, *T. heterocerus* and *T. obscurator*) are known to hibernate as diapausing adults inside host pupae in the soil (Ulber et al. 2010). After emergence in spring, individuals disperse to the surrounding landscape to locate new OSR fields. Therefore, the closest distance between the OSR field of the previous year and OSR fields of the study year might be a relevant predictor for the composition of parasitoid and host communities. For example, colonization of OSR fields by pollen beetle parasitoids can be enhanced by growing OSR as close as possible to previous year OSR locations (Hokkanen et al. 1988; Tscharntke 2000).

Given the complex relationships between agricultural landscapes and multi-species communities in oilseed rape, the aim of this study was to analyse the species compositions of multiple host–parasitoid communities sampled during one oilseed rape season in southern Sweden in 2010. The resulting data were then used to test how the variation in species composition of both trophic levels and the topologies of their interaction networks is affected by selected landscape characteristics. Specifically, we tested the hypothesis (1) that variation in species composition and network topology depends on proximity of hibernation sites (woody areas for hosts, and places where OSR was grown the previous year for parasitoids). Based on this hypothesis, we expected that (a) proximity of woody areas affects the host species number positively in contrast to weak effects on parasitoid species number, and (b) proximity of previous year OSR affects the parasitoid species number positively in contrast to weak effects on host species number. This should lead to a more pronounced asymmetry of interaction networks in OSR fields that are (a) far away from woody areas and (b) close to previous year OSR. Further, we tested the hypothesis (2) that the variation in species composition and network topology depends on habitat area (expressed as proportion of OSR in the surrounding landscape). We expected that (a) habitat area in the study year should influence the species composition of hosts and (b) habitat area of the previous year should influence the species composition of parasitoids.

## Materials and methods

### Study sites and sampling

**Fig. 1:**
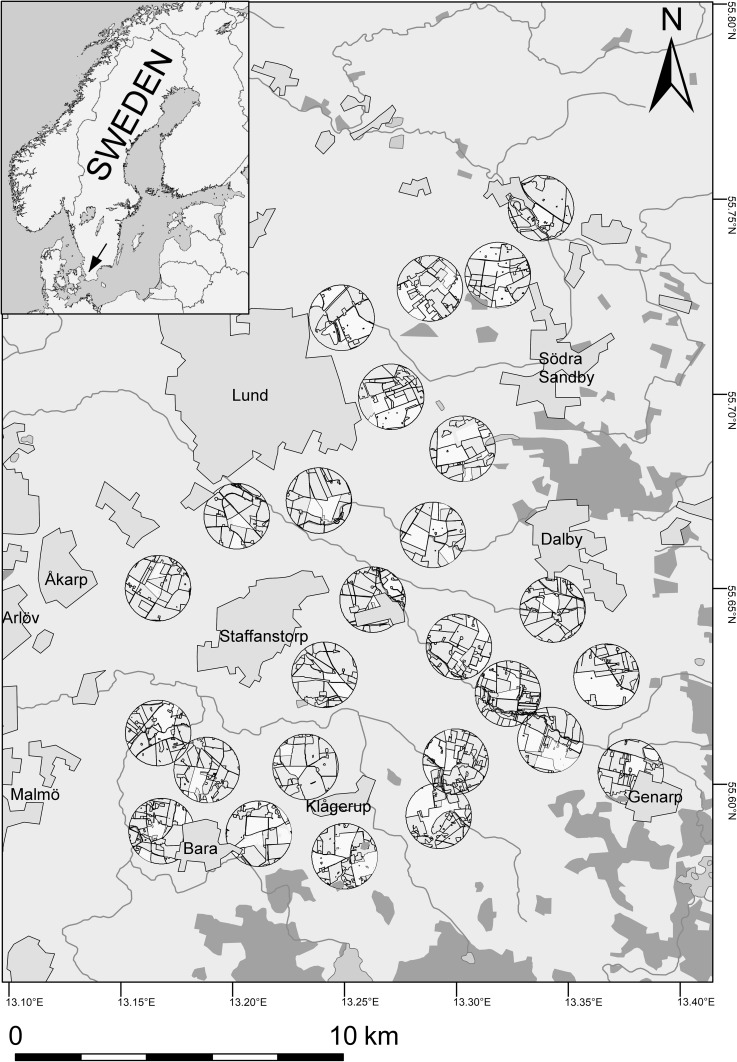
Sample sites in the agricultural landscape near Lund in southern Sweden. Around each sampling site, circles with 1 km radius are drawn and field borders indicated within these circles. Blue = rivers and water areas, green = mixed deciduous forest areas, grey = urban areas, light brown = agricultural area, yellow = winter oilseed rape area within 1 km distance from the sampling site. The black arrow in the inlay figure indicates the position of the study region in southern Sweden. (Color figure online)

The study sites consisted of 26 oilseed rape (OSR) fields grown by local farmers in a landscape with a high proportion of arable land (mean and SD 85.52 ± 7.39% within a 1000-m radius around each site) and highly intensive agricultural management, situated between Malmö and Lund in southern Sweden (Fig. 1). At all sites, winter oilseed rape was grown in a similar way in crop rotation which is common in this part of Sweden: barley > winter oilseed rape > winter wheat > sugar beet, with reduced tillage applied to cereals and tillage to sugar beet. Sites were selected according to their distance from previous year OSR which was either directly adjacent (in a distance of 30 m from the site) or not adjacent (ranging between 375 and 1061 m), and according to a variable proportion of OSR within a radius of 1000 m around each sampling site (between 0.0 and 33.5% in 2009, and between 3.3 and 30.9% in 2010). The radius of 1000 m was chosen because landscape sectors of 1–2 km diameter have previously been suggested as important spatial scale for the dispersal of OSR parasitoids (Bianchi et al. 2008; Thies and Tscharntke 2010). The agricultural area data were obtained from the Integrated Administration and Control System of the Swedish Board of Agriculture. The landscape proportions and distances were calculated with ArcGIS software 9.3.

Insects were sampled from 11 May to 21 June 2010 in water traps which collected insects continuously (i.e. 24 h every day). During this period, the oilseed rape plants developed from the bud stage to seedpod maturation. However, since not all traps could be placed simultaneously in all 26 fields during the first days of trapping, the first collection week (when OSR fields were exclusively in the bud stage) was excluded from the subsequent analyses. The resulting standardized sampling effort thus represents a continuous sampling time of 30 ± 2 days (mean ± SD) from the onset of flowering to seedpod maturation.

Traps were placed into the OSR field at a distance of 30 m from a field edge not bordering another OSR field in the study year, and separated by approximately 20 m from each other. For the analysis, we pooled catch data from three traps per site (thus corresponding to a total catch surface of 0.124 m^2^) over the standardized collection period. At each site, insects were collected with transparent plastic trays (capturing a surface of 18 cm × 23 cm = 414 cm^2^) which were placed on the soil and filled with a watery solution of benzoic acid and some drops of detergent to reduce surface tension. The collected insects were emptied from the trap once a week, and the water tray was refilled as described above. Such water traps are commonly applied to sample coleopterous pests of oilseed rape (Williams et al. 2003), but the trays capture many other arthropods, including parasitoid wasps and a variety of herbivores. Although water trap catches, due to their location on the soil, can be biased towards arthropods that are active in lower strata of the vegetation, it is a simple and standardized method to collect insects continuously during the entire time period at all 26 sites.

After collection, all insects were transferred into 70% ethanol for storage.

### Species identification

Herbivorous Coleoptera and Heteroptera and parasitoid Hymenoptera were air-dried, card-mounted and identified to species level using the most recent taxonomic literature and by comparison to reference specimens at the Entomological collection of the Zoological Museum in Lund, Sweden. Identification of selected specimens was verified by taxonomists working in the same museum (see Acknowledgements). Aphids and Diptera were excluded from the analysis due to constraints in taxonomic expertise and lack of resources.

In this study, we were interested in the herbivore–parasitoid communities associated with oilseed rape as host plant. Consequently, we have a priori included only those species of Coleoptera and Heteroptera that are known to feed on Brassicaceae during some part of their life cycle, either as adults, larvae or during both stages (Online Resource 1). Likewise, we included only those species of parasitoid Hymenoptera that can be associated with the identified herbivores based on published host records (Online Resource 1).

### Landscape characteristics

We used the following landscape characteristics as potential predictors of the occurrence of parasitoid and host species in OSR fields and their potential interactions: proportion of OSR in a 1000-m radius around the traps in the study year (OSR_*t*_) and in the previous year (OSR_*t*−1_), distance to the closest OSR field in the previous year and distance to the nearest forest edge. Spatially explicit data on where OSR was grown in the previous year (2009) and in the study year (2010) were obtained from the yearly updated database of the Integrated Administration and Control System maintained by the Swedish Board of Agriculture, in which all registered Swedish farmers report which crops and other land use types they have each year on their farmland. The basic unit of this system is an “agricultural block”, which consists of one or several adjacent fields that belong to the same farmer and are delimited by borders that can be identified from aerial photographs (such as stone walls, roads, buildings and forests). It is thus not possible to extract individual field size from the database, but the IACS has information on the relative area covered by individual crops within each block. This database information was complemented by extensive field surveys in both 2009 and 2010. According to a methodological report of the Swedish Board of Agriculture to the Food and Agriculture Organization of the United Nations, the quality on the crop area information in IACS is very high since there are regular controls and farmers may lose their EU subsidies if they report incorrect crop area (Jordbruksverket 2010: p. 23).

The surrounding forests are mixed deciduous (mainly consisting of *Fagus sylvatica*, *Quercus robur* and *Betula pendula*) with interspersed planted *Picea abies* and *Pinus sylvestris*, all grown for economic purpose. Spatially explicit data on forest area were obtained from the general map of the study region provided by the Swedish National Land Survey (Lantmäteriet) which does not differentiate between plant species compositions of areas within the forest category. Nevertheless, this map is very accurate as it is based on both satellite images and aerial photography, with the latter having a resolution of 1 pixel corresponding to 0.5 m (Lantmäteriet 2015: p. 5). Distances between each sampling site and nearest forest margin were obtained by calculating a distance matrix between a central node at each site and the nearest target node of every forest polygon.

Based on a questionnaire filled in by the local farmers, we know that 8 of the 26 oilseed rape fields were treated with insecticides (5 fields with pyrethroids, 1 field with neonicotinoids and 1 field with both) in late April/early May 2010 and 13 were not treated with insecticides. The difference in community composition between these 8 sprayed and 13 unsprayed sites was not significant (PERMANOVA, *n* = 21, pseudo-*F*_1,19_ = 1.22, *p* = 0.311). Unfortunately, it was impossible to obtain information on insecticide status for the remaining five sites; therefore, this variable was excluded from the set of predictors in our distance-based linear models for all 26 sites. Visual inspection of scatterplots did not reveal any dissimilarity pattern that would distinguish the five sites with unclear insecticide regime from the 21 sites with known insecticide regime.

The landscape composition metrics were tested for spatial autocorrelation using an inverse Euclidean distance matrix that was calculated from latitude and longitude coordinates of each site, and the function Moran. I in the R package ape (Paradis et al. 2004). We found no evidence for the presence of spatial autocorrelation in all metrics except for forest distance, whose Moran’s I autocorrelation coefficient was very low but significantly different from zero (Moran’s *I* = 0.092, SD = 0.035, *p* = 0.0002). Its positive sign indicates that neighbouring sites have similar distances to the nearest forest, which makes sense given the distribution of forests in the study landscape (see Fig. 1).

### Analysis

To identify relationships between landscape characteristics and the composition of communities, we transformed tables of host or parasitoid species abundances at each site into resemblance matrices between sites using the Sørensen similarity measure for the presence/absence data.

To test effects of landscape characteristics on the topology of the trophic networks, we generated bipartite host–parasitoid matrices between the species observed at each site, with *m* rows (host species) and *n* columns (parasitoids). Links (potential interactions) between observed host and parasitoid species were added to the matrix based on literature records (Online Resource 1). From these bipartite matrices, we calculated two qualitative food web metrics using the *bipartite* package in R (Dormann et al. 2008, 2009): (1) web asymmetry describes the discrepancy between the number of interacting higher and lower trophic level species observed at each site, which is an important feature in the robustness of bipartite networks to extinctions (Pastor et al. 2012; Santamaría et al. 2014). Adapting the formula from Blüthgen et al. (2007) to parasitoid–host systems, web asymmetry is calculated as *W* = (*P* − *H*)/(*P* + *H*) with *P* = the number of parasitoid species, and *H* = the number of host species. This yields values within the interval [− 1; 1], with positive values indicating more parasitoid than host species in the network, while the opposite is the case with negative values. (2) Qualitative linkage density (called “Link per species” in the *bipartite* package) measures the extent to which nodes in a network are connected to each other, indicating the complexity of the network (Miranda et al. 2013). It is calculated as the number of trophic links divided by the number of species in each matrix. This index indicates the diversity of interactions, which is directly analogous to species diversity (Tylianakis et al. 2010).

To test for relationships between landscape characteristics and parasitoid or host community composition and network metrics, we used a multiple regression approach that was developed to analyse multivariate community data. Distance-based redundancy analysis (db-RDA) is a multivariate ordination technique that tests for the proportion of variance in species composition that can be predicted from a linear combination of predictor variables. These analyses are based on a chosen distance matrix that is subjected to a principal coordinate analysis followed by a series of multiple regressions and a principal component analysis, using nonparametric permutation methods (Legendre and Anderson 1999; Anderson et al. 2008). For selection of the most parsimonious model we used the Akaike information criterion (AIC) and a stepwise selection procedure that combines forward and backward selection.

To be able to compare community matrices of different sizes with focus on true species turnover and not on species richness, we partialled out the effects of overall matrix size prior to fitting landscape predictors in the analyses of host and parasitoid species composition. All statistical analyses were done in PRIMER 6.0 (Clarke and Gorley 2006) with PERMANOVA+ add-on.

## Results

### Distribution of species in the local communities

At all 26 sites together and a total trap surface of 3.2292 m^2^, we identified 13,818 herbivore host individuals from 17 species that can be associated with Brassicaceae, and 4076 parasitoid individuals from 21 species that can be associated with these hosts. All species are listed in Online Resource 1. The host communities of the 26 OSR fields were dominated numerically by the rape pollen beetle *B. aeneus*, the cabbage stem weevil *C. pallidactylus* and the common weevil *Ceutorhynchus typhae*, and parasitoid communities were dominated by *Tersilochus obscurator*, *Stibeutes curvispina* (both parasitoids of the cabbage stem weevil) and *T. heterocerus* (parasitoid of the pollen beetle). These very common species were collected in all fields and show therefore no variation in the presence/absence patterns.

### Insect community composition and landscape factors

**Fig. 2:**
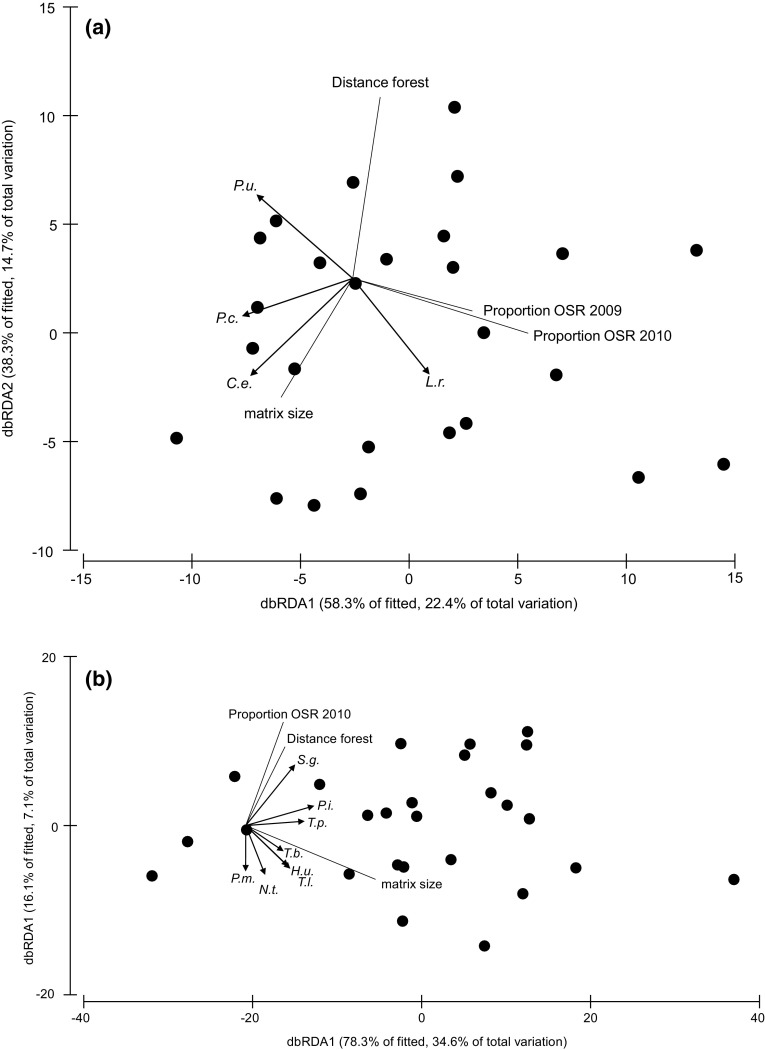
Ordination plots showing the relationship between landscape predictors and species composition of **a** host and **b** parasitoid communities in OSR fields. The axes represent linear combinations of the original variables (incidences of 17 host species in panel a, or 21 parasitoid species in (**b**). The first axis (db-RDA1) explains most of the variation in the data matrix, and the second axis (db-RDA2) explains most of the remaining variation after the first axis has been extracted but is uncorrelated with the first. Black dots show the communities at individual sites, lines indicate the direction of the effect of selected predictors, and arrows show species that were correlated with db-RDA axis scores with a Spearman coefficient > 0.4. Species abbreviations are: **a** herbivore species: C.e. = *Ceutorhynchus erysimi;* L.r. = *Lygus rugulipennis*; P.u. = *Phyllotreta undulata;* P. c. = *Psylliodes chrysocephala*. **b** Parasitoid species: H.u. = *Hemiptarsenus unguicellus*; N.t. = *Necremnus tidius*; P.m. = *Phradis morionellus*; P.i. = *Phradis interstitialis*; S.g. = *Stenomalina gracilis*. T.l. = *Trichomalus lucidus*; T.p. = *Trichomalus perfectus*

In total, 31% of the variation in host community composition was explained by a linear combination of three landscape parameters: OSR_*t*_ proportion, forest distance, and OSR_*t*−1_ proportion (ΔAIC = 1.4; *R*^2^ = 0.31; Table 1a). The occurrence of a few herbivore species accounts for most of this variation (Fig. 2a): landscapes that were located closer to a forest were characterized by the occurrence of *Psylliodes chrysocephala* and *Ceutorhynchus erysimi*. The proportion of OSR_*t*_ and OSR_*t*−1_ was positively related to the occurrence of *L. rugulipennis* and negatively related to the occurrence of *Phyllotreta undulata* (Table 2a; Fig. 2a).

**Table 1:** Predictors selected in the most parsimonious distance-based linear models for (a) host and (b) parasitoid community composition, and (c) web asymmetry and (d) linkage density in networks of potential parasitoid–host interactions

Response data	Selected predictors	*R* ^2^	*p*	Models
(a) Host communities	1. Proportion of OSR_*t*_	0.12	0.021	2/2
	2. Distance to nearest forest	0.10	0.028	2/2
	3. Proportion of OSR_*t*−1_	0.09	0.044	1/2
(b) Parasitoid communities	1. Distance to nearest forest	0.07	0.032	5/5
	2. Proportion of OSR_*t*_	0.07	0.022	3/5
(c) Web asymmetry	1. Distance to nearest forest	0.35	0.001	3/3
	2. Distance of OSR_*t*−1_	0.10	0.058	3/3
(d) Linkage density	1. Distance to nearest forest	0.14	0.065	5/5
	2. Proportion of OSR_*t*_	0.13	0.057	4/5

The last column shows the number of alternative models within 2 ΔAIC values that included the selected predictor and the total number of alternative models

OSR_*t*_ = oilseed rape in the study year, OSR_*t*−1_ = oilseed rape in the previous year

**Table 2:** Average values of landscape predictors for species that were correlated with axis scores of the distance-based redundancy analysis with a Spearman coefficient *r*_s_ > 0.4

Species	Predictor	Average value of predictor in the presence of species	Average value of predictor in the absence of species
(a) Hosts			
*Ceutorhynchus erysimi*	Forest distance	1673 m	3347 m
*Ceutorhynchus erysimi*	Proportion of OSR_*t*_	0.147	0.136
*Ceutorhynchus erysimi*	Proportion of OSR_*t*−1_	0.089	0.121
*Lygus rugulipennis*	Forest distance	113.5 m	248 m
*Lygus rugulipennis*	Proportion of OSR_*t*_	0.191	0.125
*Lygus rugulipennis*	Proportion of OSR_*t*−1_	0.147	0.097
*Phyllotreta undulata*	Forest distance	3258 m	2409 m
*Phyllotreta undulata*	Proportion of OSR_*t*_	0.099	0.162
*Phyllotreta undulata*	Proportion of OSR_*t*−1_	0.086	0.121
*Psylliodes chrysocephala*	Forest distance	2467 m	2828 m
*Psylliodes chrysocephala*	Proportion of OSR_*t*_	0.133	0.144
*Psylliodes chrysocephala*	Proportion of OSR_*t*−1_	0.058	0.136
(b) Parasitoids			
*Hemiptarsenus unguicellus*	Forest distance	3374 m	2581 m
*Hemiptarsenus unguicellus*	Proportion of OSR_*t*_	0.132	0.142
*Necremnus tidius*	Forest distance	241 m	215 m
*Necremnus tidius*	Proportion of OSR_*t*_	0.112	0.143
*Phradis interstitialis*	Forest distance	3320 m	1717 m
*Phradis interstitialis*	Proportion of OSR_*t*_	0.133	0.152
*Phradis morionellus*	Forest distance	2003 m	3014 m
*Phradis morionellus*	Proportion of OSR_*t*_	0.138	0.141
*Stenomalina gracilis*	Forest distance	3307 m	2099 m
*Stenomalina gracilis*	Proportion of OSR_*t*_	0.155	0.125
*Trichomalus lucidus*	Forest distance	2509 m	2967 m
*Trichomalus lucidus*	Proportion of OSR_*t*_	0.149	0.128
*Trichomalus perfectus*	Forest distance	2849	2216
*Trichomalus perfectus*	Proportion of OSR_*t*_	0.144	0.127
*Townesilitus bicolor*	Forest distance	2569 m	2708 m
*Townesilitus bicolor*	Proportion of OSR_*t*_	0.170	0.139

OSR_*t*_ = oilseed rape in the study year, OSR_*t*−1_ = oilseed rape in the previous year

In total, 14% of the variation in parasitoid community composition was explained by a combination of forest distance and OSR_*t*_ proportion (ΔAIC = 0.4; *R*^2^ = 0.14; Table 1b). The occurrence of multiple parasitoid species accounted for most of this variation (Fig. 2b): landscapes that were located closer to forest and had lower proportions of oilseed rape in the study year (OSR_*t*_) were characterized by the occurrence of *Phradis morionellus* and *Necremnus tidius*. Landscapes further away from forests with more oilseed rape in the study year were characterized by the occurrence of *Stenomalina gracilis* (Table 2b; Fig. 2b).

### Interaction networks and landscape factors

**Fig. 3:**
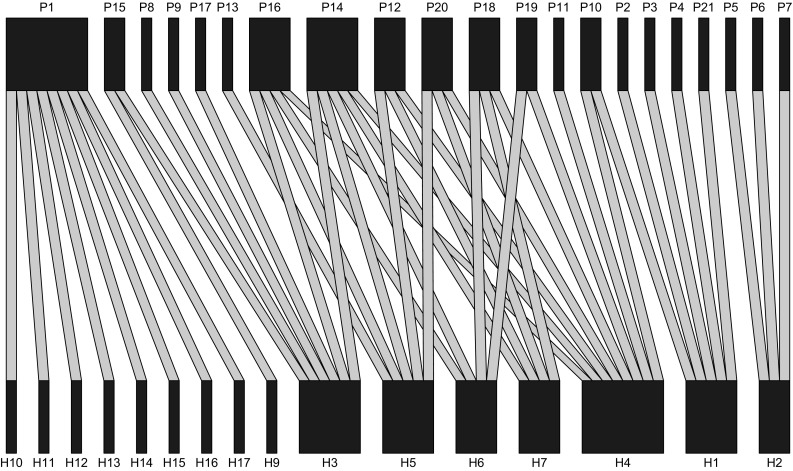
Metacommunity structure of herbivores and parasitoids collected in 26 winter oilseed rape fields in southern Sweden. The upper row represents parasitoid species; the lower row represents host species. The grey lines indicate potential trophic links inferred from host records in the literature. The coding of species identities and the literature used to infer potential trophic links are given in Online Resource 1

The bipartite interaction web in Fig. 3 shows the potential interaction links between all species in the metacommunity. Ten out of 21 parasitoid species have more than one host species (ranging between 2 and 4 hosts), while the remaining 11 parasitoid species have only 1 potential host species each. Seven of the host species had more than one potential parasitoid species (ranging from 3 to 8 parasitoids). The flea beetles (*P. chrysocephala*, *Chaetocnema concinna* and all 7 *Phyllotreta* species) had only 1 potential link each. The turnip gall weevil *Ceutorhynchus sulcicollis* was the only beetle species without any interaction link because none of its known parasitoids was caught; hence, it is not shown in Fig. 3.

**Fig. 4:**
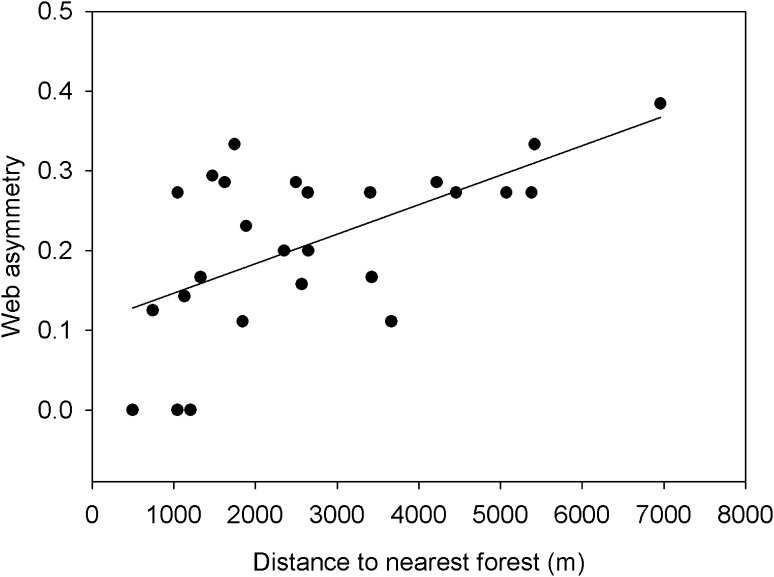
Relationship between web asymmetry and distance to the nearest forest (Pearson *R* = 0.60; *N* = 26; *p* = 0.001)

Out of the 26 networks of potential interactions between parasitoids and hosts, 3 had an equal number of taxa from both trophic levels and 23 were asymmetric, all with more parasitoid species than host species being present. In total, 45% of the variation in web asymmetry was explained by a combination of forest distance and distance to OSR_*t*−1_ (ΔAIC = 0.7; *R*^2^ = 0.45; Table 1c). Increasing forest distance led to a higher number of parasitoid species than host species (Fig. 4: Pearson *R* = 0.60; *N* = 26; *p* = 0.001). The residuals of this correlation (i.e. the variation in web asymmetry that was not explained by forest distance) showed a weak negative linear relationship with distance to OSR_*t*−1_ (Pearson *R* = − 0.35; *N* = 26; *p* = 0.079).

The average qualitative linkage density was 0.9 ± 0.2 (mean and SD). In total, 27% of the variation in qualitative linkage density was explained by a combination of forest distance and proportion of OSR_*t*_ (ΔAIC = 19.9; *R*^2^ = 0.27), but neither of these predictors was significantly correlated with this metric (Table 1d). The relationship between qualitative linkage density with forest distance was weak and not significant (Pearson *R* = 0.31; *N* = 26; *p* = 0.123), and neither was the relationship between residual linkage density and the proportion of OSR_*t*_ (Pearson *R* = 0.13; *N* = 26; *p* = 0.527).

## Discussion

Our study highlights that the community composition of herbivorous hosts and parasitoids in oilseed rape fields and their potential interaction networks strongly depend on multifaceted aspects of landscape composition.

### Community composition

Our expectations 1.a and 2.a were met for both trophic levels, supporting the hypothesis that community composition is affected by (1) forest distance and (2) habitat area. Contrary to our expectation 1.b, the shortest distance between sampling sites and the oilseed rape field of the previous year was not selected as predictor of variation in the species composition of neither trophic level. For the species composition of the parasitoid community which includes species that hibernate in previous year OSR fields, it is thus irrelevant whether the latter are 30 or 1061 m away from the sampling site. A likely explanation is that this distance range is sufficiently small to be easily overcome by flight ability of the parasitoid species. All species in our study are capable of flight, with the exception of the brachypterous *Eupelmus vesicularis* that was caught at only one site. The latter species is a polyphagous generalist with more than 200 host species in six insect orders (Noyes 2016), not confined to the OSR habitat.

The incidence of three beetle species (*P. undulata*, *P. chrysocephala*, and *C. erysimi*) was negatively related to the proportion of OSR in the surrounding, which suggests that these species profit from other habitats within the area. For the flea beetle species, alternative agricultural brassicaceous crops may be more attractive than oilseed rape (Metspalu et al. 2014). For the oligophagous weevil *C. erysimi*, whose larvae have been reported to occur simultaneously with those of *C. pallidactylus* in petioles and stems of *Brassica napus* ssp. *rapifera* in Denmark (Günthart 1949), the preferred host plant is assumed to be *Capsella bursa*-*pastoris* (Rheinheimer and Hassler 2013); hence, this species is likely to occur in any habitat where this wild brassicaceous weed is growing. This weed is common in our study region and is also supposed to be the preferred feeding plant for another oligophagous weevil, *C. typhae*, which was found in all sites of this study in high abundances. Since many other pests may be harboured by weeds, we recommend that future studies on community composition take particularly into account the effect of weed plants in both crop areas and non-crop areas such as field margins and fallows.

We found that the incidence of *P. morionellus* in OSR fields was supported by forest proximity, while the opposite was found for its sibling species *P. interstitialis*. This may add an additional dimension to the niche separation between these two *Phradis* species (Berger et al. 2015). Both are parasitoids of the rape pollen beetle. *P. morionellus* is also frequently encountered in spring oilseed rape grown in central Sweden and in Finland (Billqvist and Ekbom 2001; Hokkanen 2006), regions that are more densely covered by forest compared to our study region. Our result suggests that *P. morionellus* may during some part of its life cycle benefit from forest proximity, possibly by exploiting alternative hosts in the forest habitat. So far, the host range of this parasitoid outside oilseed rape is poorly known, but includes at least four additional pollen beetle species that feed on other plants than *B. napus* (Khalaim et al. 2009). Overwintering in alternative hosts outside OSR would enhance the ability of this parasitoid species to persist in the changing agricultural landscape and colonize new OSR habitats.

Interestingly, *S. gracilis* was mainly observed in OSR fields that were further away from forests and had a high proportion of host habitat in the study year. The incidence of this species was not related to any local or landscape-scale predictors when sampled from thistles in wheat fields (Clough et al. 2007); however, this contrast may be due to the different methodologies and different study systems. This species, which recently was identified as one of the key species for conservation biocontrol of oilseed rape pests (Ulber et al. 2010), is either a broad generalist (as indicated by 28 host records from 4 insect orders in Noyes 2016) or a conglomerate of several cryptic species that all look the same but are specialized on different hosts. It would be crucial to clarify the species status with molecular methods using material obtained from different hosts, to improve our knowledge about host specificity at the species level and about trophic links at the community level (Hrček and Godfray 2014; Wirta et al. 2014).

### Potential interaction networks

We found that the topology of the interaction network, as indicated by web asymmetry, was affected primarily by forest distance, which supports our expectation 1.a that proximity of forests affects host species number positively in contrast to weak effects on parasitoid species number. Our potential interaction webs became increasingly asymmetric with increasing forest distance, due to relatively fewer host than parasitoid species. Several host species in our study hibernate under leaf litter in woodland (Nilsson 1989; Alford et al. 2003; Williams 2010). Forest proximity thus supports colonization of nearby agricultural fields by a larger number of host species, whereas sites in larger distances from forests get colonized by less host species. The increase in web asymmetry with forest distance can also be interpreted the other way around—as negative effect of forest proximity on the number of parasitoid species, leading to relatively more parasitoid species than host species in sites that are far away from forests. A potential explanation might be the wind barrier hypothesis (Kaasik et al. 2014): since the host detection of parasitoids is highly dependent on wind flow (Williams et al. 2007), nearby forests might act as wind barrier for parasitoid dispersal or for their detection of host odours, whereas larger distance from forests may enable parasitoids to be more efficient in detecting host odours and therefore increase their colonization probability. Growing OSR preferably in landscapes that are far away from forests may therefore be a suitable strategy both to minimize recolonization by pest species and to attract a higher number of parasitoid species from the open landscape.

### Limitations of the study

One of the limitations of our study is that it is exclusively based on incidences (i.e. detected presence/absence information) of parasitoid and host species, and consequently only a qualitative network analysis of potential interactions was possible. This is a conservative approach to community and interaction analyses that may underestimate relationships to landscape characteristics and that emphasizes the role of rare species. We chose this approach because our data come from trap catches; hence, we have no unbiased measure of abundance or direct measure of interaction strength for a quantitative analysis. To infer interaction strength from species abundances in the traps would require several additional assumptions, such as equal representation of species abundances in the traps for all taxa; however, insects that are active in the lower strata of vegetation are likely to be more abundant in the traps than insects active in the canopy. A direct estimation of trophic interactions would have required different sampling techniques and laboratory methods (e.g. rearing, or molecular analysis) which in our case were impractical due to the large number and taxonomic range of parasitoid and host species.

A further limitation is that we have only tested landscape effects connected to one crop plant, since we were primarily interested in the tritrophic system oilseed rape—parasitoids–hosts. However, the incidence of several polyphagous generalists is likely to depend on more than one plant species in the landscape. A different study design would be needed to elucidate how much the community composition of parasitoids and their hosts in oilseed rape are affected by weed plants or sown wildflowers, as these may support several insect species by the provision of food, shelter and alternative hosts (Rusch et al. 2010).

### Conclusions

Our results show that the configuration of agricultural landscape affects both the maintenance of species composition and the architecture of potential interaction networks. The distance to forest elements in the landscape and the proportion of habitat area play important roles in explaining variation in both the host and the parasitoid community composition, and it affects the topology of the interaction network by a shift towards less pest species and more parasitoid species. As a practical implication of this study, we predict that growing oilseed rape fields in regions far away from forests is likely to minimize the recolonization by pest species and to attract a higher species richness of their natural enemies. This adds to a growing amount of evidence that a landscape perspective is valuable for our understanding of parasitoid–host relationships, since certain habitat types can be seen as relevant immigration sources of pests and parasitoids. How species composition of parasitoid communities translates to biological control of oilseed rape pests is, however, largely unknown since biological control studies of this ecosystem have so far only focused on the abundance of one or a few of the most abundant key species. More research is needed to elucidate the causal cascades between community composition and crop production, notably by the analysis of crop yield as response variable.

### Author contribution statement

HH selected the landscapes and some predictors and began the field work. JB did most of the field work, all laboratory work and all species identifications, assembled the linkage matrix and selected additional predictors. KB did the statistical analysis. KH was involved in the conception and funding of the study and provided the laboratory facilities. JB wrote the manuscript. KB, KH and HH contributed to the final version of the manuscript.

## Electronic supplementary material

Below is the link to the electronic supplementary material.

Supplementary material 1 (PDF 722 kb)
