# Supplementary material for: Landscape configuration affects herbivore–parasitoid communities in oilseed rape
Source: J Pest Sci (2004). 2018 Feb 24;91(3):1093–105. doi: 10.1007/s10340-018-0965-1 (PMC5978837; doi:10.1007/s10340-018-0965-1)
Supplement: Supplementary file 1 — Supplementary material 1 (PDF 722 kb) [file 10340_2018_965_MOESM1_ESM.pdf]

Landscape configuration affects herbivore-parasitoid communities in oilseed rape (Supplementary Information)

Josef S. Berger[1], Klaus Birkhofer[1,2,3], Helena I. Hanson[2], Katarina Hedlund[1,2]

[1] Department of Biology, Lund University, Sölvegatan 37, SE-223 62 Lund, Sweden

[2] Centre for Environmental and Climate Research, Lund University, Sölvegatan 37, SE-223 62 Lund, Sweden

[3] Department of Ecology, Brandenburg University of Technology Cottbus-Senftenberg, Konrad-Wachsmann-Allee 6, 03046 Cottbus, Germany

Corresponding author:

Josef S. Berger

josef.berger@biol.lu.se

+46462228003

Supplementary Information Table 1:

Literature used to infer feeding preferences of herbivores (the code column indicates the species identity in Figure 3).

Code	Herbivore	Host range	Adult feeding niche	Larval feeding niche	References
	**Heteroptera: Miridae**				
H1	*Lygus rugulipennis*	highly polyphagous	stems, buds, flowers and pods	same as adult	Rämert et al. 2005
	**Coleoptera: Nitidulidae**				
H2	*Brassicogethes aeneus (syn. Meligethes aeneus)*	polyphagous on Brassicaceae	buds and flowers	pollen in buds & flowers	Williams 2010
	**Coleoptera: Curculionidae**				
H3	*Ceutorhynchus pallidactylus (syn. C. quadridens)*	oligophagous on Brassicaceae	leaves	leaf and stem miner	Koch 1992; Morris 2008
H4	*Ceutorhynchus obstrictus (syn. C. assimilis)*	polyphagous on Brassicaceae	buds, flowers, leaves	seed pods	Koch 1992; Morris 2008
H5	*Ceutorhynchus typhae (syn. C. floralis)*	oligophagous on Brassicaceae	leaves	seed pods	Koch 1992; Toshova et al. 2009
H6	*Ceutorhynchus contractus*	polyphagous on Brassicaceae	leaves	leaf miner	Koch 1992; Morris 2008
H7	*Ceutorhynchus erysimi*	polyphagous on Brassicaceae	leaves	leaf and stem miner	Koch 1992; Morris 2008
H8	*Ceutorhynchus sulcicollis*	polyphagous on Brassicaceae et al.	leaves	leaf and stem miner	Koch 1992; Morris 2008
	**Coleoptera: Chrysomelidae**				
H9	*Psylliodes chrysocephala*	Brassicaceae	leaves	leaf and stem miner	Rufelt 1995
H10	*Chaetocnema concinna*	polyphagous on Brassicaceae et al.	leaves and pollen	root hairs	Augustin et al. 1986, Metspalu et al. 2014
H11	*Phyllotreta atra*	polyphagous on Brassicaceae et al.	cotyledons, stems, young leaves	root hairs	Günthart 1949; Augustin et al. 1986; Soroka 2008
H12	*Phyllotreta flexuosa*	polyphagous on Brassicaceae et al.	cotyledons, stems, young leaves	root hairs	Günthart 1949; Augustin et al. 1986; Soroka 2008
H13	*Phyllotreta nemorum*	polyphagous on Brassicaceae et al.	cotyledons, stems, young leaves	leaf miner	Ekbom 2010
H14	*Phyllotreta striolata*	polyphagous on Brassicaceae et al.	cotyledons, stems, young leaves	root hairs	Günthart 1949; Augustin et al. 1986; Soroka 2008
H15	*Phyllotreta tetrastigma*	polyphagous on Brassicaceae et al.	cotyledons, stems, young leaves	root hairs	Günthart 1949; Augustin et al. 1986; Soroka 2008
H16	*Phyllotreta undulata*	polyphagous on Brassicaceae et al.	cotyledons, stems, young leaves	root hairs	Günthart 1949; Augustin et al. 1986; Soroka 2008
H17	*Phyllotreta vittula*	polyphagous on Brassicaceae et al.	cotyledons, stems, young leaves of Brassicaceae	leaf and stem petioles of cereals	Ekbom 2010

Supplementary Information Table 2:

Literature used to infer host range of parasitoids (the code column indicates the species identity in Figure 3).

Code	Parasitoid	Mode of parasitism	Host range	References
	**Braconidae: Euphorinae**			
P1	*Townesilitus bicolor*	koinobiont adult endoparasitoid	broad generalist; wide host record on many Chrysomelidae Alticinae, incl. *Phyllotreta* and *Chaetocnema* spp.	Tobias 1995; Cox 1994
P2	*Peristenus digoneutis*	koinobiont		
P3	*Peristenus relictus*	nymphal	broad generalists; wide variety of tribes and subfamilies of	Rämert et al. 2005; Mason et al. 2011
P4	*Peristenus pallipes*	endoparasitoids	Miridae (Heteroptera), including *Lygus rugulipennis*.	
	**Ichneumonidae: Tersilochinae**			
P5	*Phradis interstitialis*	koinobiont egg-larval endoparasitoid	*Brassicogethes aeneus* and other *Brassicogethes* spp.	Khalaim et al. 2009; Ulber et al. 2010
P6	*Phradis morionellus*	koinobiont larval endoparasitoid	*Brassicogethes aeneus* and other *Brassicogethes* spp.	Khalaim et al. 2009; Ulber et al. 2010
P7	*Tersilochus heterocerus*	koinobiont larval endoparasitoid	*Brassicogethes aeneus* and *viridescens*	Khalaim et al. 2009; Ulber et al. 2010
P8	*Tersilochus obscurator*	koinobiont larval endoparasitoid	*C. pallidactylus*	Ulber et al. 2010
	**Ichneumonidae: Cryptinae**			
P9	*Stibeutes curvispina*	idiobiont pupal / prepupal ectoparasitoid	*C. pallidactylus*	Horstmann 2010
	**Mymaridae: Mymarinae**			
P10	*Anaphes fuscipennis*	idiobiont egg parasitoid	broad generalist; recorded from *C. obstrictus* and *Lygus rugulipennis*	*C.obstrictus:* Ulber et al. 2010. Lygus: Rämert et al. 2005
P11	*Anaphes regulus (syn. Mymar autumnalis)*	idiobiont egg parasitoid	recorded from *C. obstrictus* and *C. contractus*	Godan 1959; Rheinheimer & Hassler 2013
	**Pteromalidae: Pteromalinae**			
P12	*Mesopolobus gemellus*	idiobiont larval ectoparasitoid	generalist on several *Ceutorhynchus* spp.; recorded from *C. erysimi*, *C. obstrictus*, and *C. typhae*	Mason et al. 2014.
P13	*Mesopolobus incultus*	idiobiont larval ectoparasitoid	wide host record, including *C. typhae*	Kuhlmann & Mason 2003
P14	*Stenomalina gracilis*	idiobiont larval or pupal ectoparasitoid	broad generalist; recorded from *C. contractus*, *C. erysimi*, *C. obstrictus*, *C. pallidactylus, C. typhae*	Gibson et al. 2006; Muller et al. 2011; Rheinheimer & Hassler 2013
P15	*Trichomalus lucidus*	idiobiont larval ectoparasitoid	several stem-boring *Ceutorhynchus* spp.; recorded from *C. pallidactylus* and *P. chrysocephala*	Muller et al. 2007; Nissen 1997
P16	*Trichomalus perfectus*	idiobiont larval ectoparasitoid	narrowly oligophagous on *Ceutorhynchus* spp; recorded from *C. contractus*, *C. obstrictus, C. typhae, C. pallidactylus*	Haye et al. 2015; Rheinheimer & Hassler 2013
P17	*Trichomalus rusticus*	idiobiont larval ectoparasitoid	*C. pallidactylus*	Muller et al. 2007
	**Eupelmidae: Eupelminae**			
P18	*Eupelmus vesicularis*	idiobiont ectoparasitoid	broad generalist; recorded from *C. erysimi*, *C. contractus, C. obstrictus*	Mason et al. 2014; Rheinheimer & Hassler 2013; Williams 2003
	**Eulophidae: Eulophinae**			
P19	*Hemiptarsenus unguicellus*	larval ectoparasitoid	broad generalist; recorded from *C. contractus* and *C. obstrictus*	Rheinheimer & Hassler 2013; Gharari & Yefremova 2013
P20	*Necremnus tidius*	larval ectoparasitoid	broad generalist; recorded from *C. erysimi*, *C. obstrictus, C. typhae*	Mason et al. 2014; Kuhlmann & Mason 2003; Ulber et al. 2010
	**Scelionidae: Telenominae**			
P21	*Telenomus sp.*	idiobiont egg parasitoid	*Lygus* bugs	Broadbent et al. 2002

References

Augustin A, Tulisalo U, Korpela S (1986): Flea beetles (Coleoptera, Chrysomelidae, Halticinae) on rapeseed and sugarbeet in Finland. *Journal of Agricultural Science in Finland* 58: 69-82.

Broadbent AB, Mason PG, Lachance S, Whistlecraft JW, Soroka JJ, Kuhlmann U (2002): *Lygus* spp., plant bugs (Hemiptera: Miridae). pp.152-159 in: Mason PG & Huber JT (eds.): *Biological Control Programmes in Canada, 1981-2000*. CABI Publishing, 583 pp.

Cox ML (1994): The Hymenoptera and Diptera parasitoids of Chrysomelidae. pp. 419-467 in: Jolivet PH, Cox ML, Petitpierre E (eds.): *Novel aspects of the biology of Chrysomelidae*. Dordrecht: Kluwer Academic Publishers, 582 pp.

Ekbom B (2010): Pests and their enemies in spring oilseed rape in Europe and challenges to integrated pest management. pp.151-165 in: Williams IH (ed.) *Biocontrol-based integrated management of oilseed rape pests*. Dordrecht, Springer: 461 pp.

Gharari H & Yefremova ZA (2013): A study of the family Eulophidae (Hymenoptera: Chalcidoidea) from Iran. *Zoosystematica Rossica* 22(2):303-310.

Gibson GAP, Gillespie DR, Dosdall L (2006): The species of Chalcidoidea (Hymenoptera) introduced to North America for biological control of the cabbage seedpod weevil, and the first recovery of *Stenomalina gracilis* (Chalcidoidea: Pteromalidae). *Canadian Entomologist* 138: pp.285-291.

Godan, D. (1959). *Mymar autumnalis* (Foerst.) (*Chalcidoidea,Mymar.*), ein bisher unbekannter Eiparasit des Kohlschotenrüsslers. *Nachrichtenblatt des Deutschen Pflanzenschutzdienstes* 11, 161–4.

Günthart, E (1949): Beiträge zur Lebensweise und Bekämpfung von *Ceuthorrhynchus quadridens* Panz. und *Ceuthorrhynchus napi* Gyll. mit Beobachtungen an weiteren Kohl- und Rapsschädlingen. *Mitteilungen der schweizerischen Entomologischen Gesellschaft* 22(5):441-588.

Haye T, Mason PG, Gillespie DR, Miall JH, Gibson GAP, Diaconu A, Brauner AM, Kuhlmann U (2015): Determining the host specificity of the biological control agent *Trichomalus perfectus* (Hymenoptera: Pteromalidae): the importance of ecological host range. *Biocontrol Science and Technology* 25:21-47.

Horstmann K (2010): Revision der europäischen Arten von *Stibeutes* Förster, 1850 (Hymenoptera, Ichneumonidae, Cryptinae). *Entomofauna* 31: pp.229-264.

Khalaim AI, Bordera S, Rodríguez-Berrío A (2009): A review of the European species of *Phradis* (Hymenoptera: Ichneumonidae: Tersilochinae), with a description of a new species from Spain. *European Journal of Entomology* 106: pp. 107-118.

Koch K (1992): *Die Käfer Mitteleuropas: Ökologie* 3. Krefeld: Goecke & Evers.

Kuhlmann U & Mason PG (2003): Use of field host range surveys for selecting candidate non-target species for physiological host specificity testing of entomophagous biological control agents. pp.370-377 in: van Driesche RG (ed.): *Proceedings of the 1st International Symposium on Biological Control of Arthropods*. Forest Service Publication FHTET-03-05. Morgantown, West Virginia: US Department of Agriculture.

Mason PG, Broadbent AB, Whistlecraft JW, Gillespie DR (2011): Interpreting the host range of *Peristenus digoneutis* and *Peristenus relictus* (Hymenoptera: Braconidae) biological control agents of *Lygus* spp. (Hemiptera: Miridae) in North America. *Biological Control* 57: pp.94-102.

Mason PG, Miall JH, Bouchard P, Brauner A, Gillespie DR, Gibson GAP (2014): The parasitoid communities associated with *Ceutorhynchus* species (Coleoptera: Curculionidae) in Ontario and Québec, Canada. *Canadian Entomologist* 146: 224-235.

Metspalu L, Kruus E, Ploomi A, Williams JH, Hiiesaar K, Jõgar K, Veromann E, Mänd M (2014): Flea beetle (Chrysomelidae: Alticinae) species composition and abundance in different cruciferous oilseed crops and the potential for a trap crop system. *Acta Agriculturae Scandinavica, Section B - Soil & Plant Science* 64(7): 572-582.

Morris MG (2008): *True weevils (part II) (Coleoptera: Curculionidae, Ceutorhynchinae).* St Albans: Royal Entomological Society, 130 pp.

Muller FJ, Baur H, Gibson GAP, Mason PG, Kuhlmann U (2007): Review of the species of *Trichomalus* (Chalcidoidea: Pteromalidae) associated with *Ceutorhynchus* (Coleoptera: Curculionidae) host species of European origin. *Canadian Entomologist* 139: 643-657.

Muller FJ, Dosdall LM, Mason PG, Kuhlmann U (2011): Larval phenologies and parasitoids of two seed-feeding weevils associated with hoary cress and shepherd's purse (Brassicaceae) in Europe. *Canadian Entomologist* 143: 399-410.

Rämert B, Hellqvist S & Petersen MK (2005): A survey of *Lygus* parasitoids in Sweden. *Biocontrol Science and Technology* 15(4): 411-426.

Rheinheimer J & Hassler M (2013): *Die Rüsselkäfer Baden-Württembergs*. (2nd ed.) Ubstadt-Weiher: Verlag Regionalkultur. 944 pp.

Rufelt S (1995): Rapsjordloppa. *Faktablad om växtskydd 54 J Jordbruk*. Sveriges Lantbruksuniversitet, 3 pp.

Soroka JJ (2008): Flea Beetles (Coleoptera: Chrysomelidae: Alticinae). pp. 1467-1474 in: Japinera JL (ed): *Encyclopedia of Entomology*. Dordrecht: Springer.

Thies C & Tscharntke T (2010): Biological rape pest control in spatio-temporally changing landscapes. pp.273-284 in: Williams IH (ed.): *Biocontrol-based integrated management of oilseed rape pests*. Dordrecht: Springer, 461 pp.

Tobias VI (1995): Family Braconidae. in: Medvedev (ed.): *Keys to the insects of the European part of the USSR Vol. 3, Hymenoptera Part IV*. New Delhi: Amerind Publishing. 883 pp.

Toshova T, Subchev M, Tóth (2009): The diversity of species of Ceutorhynchinae captured in traps in the region of Sofia, Bulgaria. *Bulletin of Insectology* 62(1): 27-33.

Ulber B, Williams IH, Klukowski Z, Luik A, Nilsson C (2010): Parasitoids of oilseed rape pests in Europe: Key species for conservation biocontrol. pp.45-76 in Williams IH (ed.): *Biocontrol-based integrated management of oilseed rape pests*. Dordrecht: Springer, 461 pp.

Williams IH (2003): Parasitoids of cabbage seed weevil. pp.97-112 in: Alford DV (ed.): *Biocontrol of oilseed rape pests*. Oxford: Blackwell Science Ltd, 355 pp.

Williams IH (2010): The major insect pests of oilseed rape in Europe and their management: an overview. pp.1-43 in Williams IH (ed.): *Biocontrol-based integrated management of oilseed rape pests*. Dordrecht, Springer, 461 pp.
